# Markedly Reduced Thermal Pain Perception in a Schizoaffective Patient with Tardive Dyskinesia

**DOI:** 10.1155/2016/8702483

**Published:** 2016-04-10

**Authors:** Stéphane Potvin, Emmanuel Stip, Serge Marchand

**Affiliations:** ^1^Centre de Recherche de l'Institut Universitaire en Santé Mentale de Montréal, 7331 Hochelaga, Montreal, QC, Canada H1N 3V2; ^2^Department of Psychiatry, Faculty of Medicine, University of Montreal, C.P. 6128, succursale Centre-ville, Montreal, QC, Canada H3C 3J7; ^3^Centre Hospitalier de l'Université de Montréal, 1560 Sherbrooke East, Montreal, QC, Canada H2L 4M1; ^4^Department of Surgery, Faculty of Medicine, University of Sherbrooke, 3001 12e avenue Nord, Sherbrooke, QC, Canada J1H 5N4

## Abstract

Several case reports have described stories of schizophrenia patients reporting no discomfort in response to several medical conditions which normally elicit pain. Comparatively, experimental studies performed on pain perception in schizophrenia have not documented hypoalgesic responses that are as frank as those reported in these clinical cases. Here, we report the case of a female patient with schizoaffective disorder, who displayed markedly reduced pain perception during an experimental heat pain paradigm. Compared to a large group of healthy volunteers that we tested in 3 previous studies using the same psychophysical procedure, the experimental temperature required to induce moderate pain was radically increased in this patient (*z*-score = 3.6). The patient had mild psychiatric symptoms and had insight into her symptoms. She had drug-induced dyskinetic symptoms. This case report illustrates that it is possible to observe marked reductions in pain perception in schizophrenia patients tested in experimental settings but that the phenomenon is relatively rare. Regardless of the exact nature of pain indifference in schizophrenia, it can delay diagnosis and treatment of medical problems in these patients. Future studies in the field will need to pay attention to drug-induced extrapyramidal symptoms.

## 1. Background

Schizophrenia patients are known for being “insensitive to pain.” In support of this widely held assumption, several case reports have described stories of schizophrenia patients reporting no discomfort in response to several medical conditions (e.g., myocardial infarction, ruptured appendix, and peritonitis) which normally elicit pain [1, 2]. However, experimental studies performed in the laboratory have not produced results that are as unequivocal as those from the clinical observations reported in these cases. Two meta-analyses have shown that pain perception is reduced in schizophrenia [3, 4]. However, the composite effect size emerged as being moderate, results were highly heterogeneous, and a minority of studies actually observed hyperalgesic responses in experimental settings in schizophrenia. In the past, we have performed an experimental study in schizophrenia patients using a thermal heat pain paradigm [5]. In that study, we found a small-to-moderate reduction in pain perception during the tonic (2-minute) heat pain stimulation. Here, we would like to report the case story of a schizoaffective disorder patient who displayed a marked reduction in heat pain perception using the same experimental paradigm. A case report like this is original in that it defines hypoalgesia based on experimental results, rather than clinical observations which maybe biased the fact that emotional expression is reduced in schizophrenia [6].

## 2. Case Presentation

The patient signed a detailed consent form approved by the local ethics committee. She is a 41-year-old woman diagnosed with schizoaffective disorder (DSM-IV-TR criteria). She suffers from the disorder since the age of 32 and has been hospitalized 6 times for psychiatric reasons. The patient has a history of suicidal ideation but no overt suicidal attempt. At the moment of being tested, however, her psychiatric symptoms are mild. As assessed with the* Positive and Negative Syndrome Scale* [7], she has few positive (PANSS-Positive: 12), negative (PANSS-Negative: 10), and general symptoms (PANSS-General: 26). She has insight into her illness (PANSS-G12: 2). As assessed with the* Calgary Depression Scale for Schizophrenia* [8], she has no depressive symptoms and no suicidal ideation (total score = 0). On tasks measuring working memory and executive functions, she has relatively few cognitive deficits compared to the other schizophrenia patients enrolled in the initial study (*z*-scores = 1.1–1.2). However, these results are based on two cognitive tasks only [5]. Her prolactin levels are within normal range: 7.9 *μ*g/L. As assessed with the* Extrapyramidal Symptoms Rating Scale* (ESRS) [9] administered by a physician trained in neurology, the patient has no parkinsonism, no akathisia, and no dystonia but has tardive dyskinesia (e.g., choreoathetoid movements, frowning of eyebrows, and eye blinking, as well as occasional buccolabial and truncal movements). She rates her dyskinesia as being mild on the subjective items of the ESRS. Based on the patient's medical file, the earliest signs of tardive dyskinesia have been observed 18 months prior to the current study. The patient is treated with clozapine (200 mg/day), which has been introduced 6 months prior to the current assessments. Prior to that, the patient has been treated with haloperidol, olanzapine, quetiapine, risperidone, and trifluoperazine. Based on her medical record, the patient is not suffering from any psychiatric, neurological, or medical condition other than schizoaffective disorder and drug-induced tardive dyskinesia (note: an EEG exam was part of the medical assessments). Finally, the patient has no history of chronic pain.

*Methods*. Thermal pain thresholds (TPTs) and tolerance (TOL) were measured by applying a 3 cm^2^ Peltier thermode (TSA II, Medoc, Advanced Medical Systems, Minneapolis, MN 55435) on the left forearm of the patient. Experimental temperature was initially set at 32°C and was gradually increased by a rate of 0.3°C per second. She was instructed to verbally report when her sensation changed from heat to pain (TPTs) and when her pain became intolerable (TOL). The procedure was repeated 2 times to ensure the stability of TPT and TOL measurements. Afterwards, a continuous heat pulse was administered with a thermode for 2 minutes on her left forearm. Experimental temperature reached a predetermined fixed value and remained constant during the 2-minute testing period. It was set at a value corresponding to a temperature individually predetermined to induce a 50/100 pain rating during the pretest (T50). During the tonic thermal pain stimulations, pain intensity was measured using a computerized visual analog scale, which ranged from 0 (no pain) to 100 (most intense pain tolerable). The tonic heat pain stimulation was administered twice. The thermode was slightly displaced between administrations to avoid peripheral sensitization.

To evaluate the patient's pain perception, we compared her pain scores to the pain scores of healthy controls tested with the same experimental procedure in our prior studies [5, 10, 11]. When relevant, we calculated *z*-scores based on the mean and standard deviation of pain outcomes.

*Results*. Pain perception was radically reduced in the patient. Her TPTs were moderately increased (48.1°C versus 43.0 ± 3.6 in controls). More strikingly, her TOL could not be determined since she was able to tolerate the maximal temperature, which was fixed at 51°C for safety reasons. Because of that, we used an experimental temperature of 50°C for the tonic heat pain stimulations, which is a temperature substantially higher than the T50 that we habitually use in healthy controls (46.8°C ± 0.9; *z*-score = 3.6). Despite the use of an extremely high T50, the patient experienced little pain during the 2-minute heat pain stimulations (mean = 20/100; *z*-score = 2), far lower than the expected 50% pain ratings, meaning that an even higher T50 would have been required to reach this target. Comparatively, only 5.5% of healthy controls had a T50 equal to or above 48.5°C (highest = 49°C; *z*-score between 1.9 and 2.4), and the mean pain perception during the 2-minute tonic heat pain stimulations was 50.9 for these individuals.

## 3. Discussion

The main conclusion of the current case report is that it is possible to observe cases of marked hypoalgesia in experimental settings in schizophrenia but that this phenomenon seems to be relatively rare. Indeed, out of 24 schizophrenia patients that were tested, the patient described here was the first to have a marked reduction in experimentally induced pain perception (*z*-score = 3.6 for the T50). The relative rarity of marked reductions in pain perception in experimental settings should not come as a surprise, considering that the clinical cases of marked pain indifference are also relatively rare [1]. As such, the current case report has methodological implications, as it implicitly implies that we should probably not expect radical differences in pain perception in schizophrenia as a* group*. In return, this raises a very difficult question: how can we identify the uncommon schizophrenia patients who are fundamentally different in that regard? To tentatively answer this question, we performed a comprehensive clinical assessment of the patient. Unexpectedly, the single factor that clearly differentiated her from the other patients enrolled in the initial study is that she suffers from drug-induced tardive dyskinesia, whereas no other patient in the initial study did so [5]. Other patients in the initial study were diagnosed with schizoaffective disorder and a history of suicidal ideation, others had a chronic course of psychosis, and others were treated with clozapine, but none of them had substantially altered pain perception [5]. To our knowledge, only one experimental study measured pain perception in patients with dyskinetic symptoms, and it showed that their pain perception was moderately reduced, relative to controls [12]. According to a leading theory, antipsychotics-induced dyskinetic symptoms are thought to result from the supersensitivity of subcortical dopaminergic receptors after prolonged exposure to these drugs [13]. Interestingly, mounting evidence shows that dopamine modifies pain perception [14]. Indeed, it has been shown that lesions of dopaminergic mesostriatal neurons abolish the antihyperalgesic effects of amphetamines in rodents [15] and that dopaminergic agonists produce analgesic effects in humans [16]. Taken together, these findings tentatively suggest that the pain perception may be reduced in schizophrenia patients with tardive dyskinesia due to common dopaminergic dysfunctions. However, it must not be forgotten that the roles of dopamine in hyperkinetic movements and pain perception remain incompletely understood [14, 15]. Moreover, the markedly reduced pain perception seen in the patient reported here could be due to several other unmeasured clinical factors. Overall, the patient did not appear as lacking insight into her psychiatric disorder and into her dyskinetic symptoms. If hypoalgesia in schizophrenia reflects a form of impaired insight, it seems to be independent from other domains of unawareness. Future studies will need to pay greater attention to drug-induced extrapyramidal symptoms when studying pain perception in schizophrenia. A very difficult challenge will lie ahead, since we need to be able to better identify the specific patients who are “pain indifferent” and the specific circumstances under which they are. It will also be important to determine if pain indifference in the laboratory is associated with pain indifference in daily life. In the current study, this was impossible to determine, since the patient had no history of medical illness known to cause significant pain. Regardless of the exact nature of this “pain indifference,” it can delay the diagnosis and treatment of physical illness in schizophrenia [1], a disorder characterized by elevated rates of early mortality [17].
